# 

**Published:** 1860-09

**Authors:** 


NORTH AMERICAN

### MEDICO-CHIRURGICAL REVIEW.

Vol. IV.] SEPTEMBER, 1860. [No. 5.

### CONTENTS.

### ANALYTICAL AND CRITICAL REVIEWS.

ART. PAGE

I.—The Diseases of the Ear. By JOSEPH TOYNBEE, F.R.S., etc. .......... 769
II.—Medical Studies on Ancient Rome. By JULES ROUYER, M.D. .......... 790
III.—Clinical Lectures on Certain Acute Diseases. By R. BENTLEY TODD, M.D. 801
IV.—On the Diseases, etc. of the Joints. By THOMAS BRYANT, F.R.S. ........ 810
V.—On Retro-Uterine Hematocle and Non-encysted Sanguineous Effusions of the Peritoneal Cavity of the Pelvis, considered as Accidents of Menstruation. By AUGUSTUS VOISIN, M.D. .......... 817
VI.—Treatise on Surgical Anatomy, etc. By J. F. MALGAIGNE, M.D. .......... 822
VII.—Pinel: A Biographical Study. By DR. CASIMIR PINEL .......... 828
VIII.—A Medico-Legal Treatise on Malpractice and Medical Evidence. By JOHN J. ELWELL, M.D. .......... 832
IX.—On the Organs of Vision. By THOMAS NUNNELEY, F.R.C.S.E. .......... 835
X.—The Anatomy of the Human Lung. By A. T. H. WATERS, M.R C.P... 837
XI.—Report of Prof. Valentine Mott's Surgical Clinics in the University of New York. By SAMUEL W. FRANCIS, M D. .......... 839
XII.—De la Circulation du Sang dans les Membres et dans la Tête chez l'Homme. Par J. F. SUCQUET, M.D. .......... 840
XIII.—The Institutes of Medicine. By MARTYN PAYNE, M.D .......... 841

### ORIGINAL COMMUNICATIONS.

I.—Dysentery; its Pathology, Causes, etc., with Cases. By H. P. AYRES, M D. 842
II.—Tracheotomy in Croup. By C. S. FENNER, M.D. .......... 854
III.—Report of a Case of Coup de Soleil, with Post-mortem Examination, and Remarks. By HUMPHREY PEAKE, M.D. .......... 859
IV.—Eclampsia in a Primipara at the Sixth Month of Utero-Gestation. By ROBERT B. S. HARGIS, M.D. .......... 862
V.—Cases of Colloid Tumor of the Abdomen. By S. D GROSS, M.D. ........ 866
VI.—Local Treatment of Gleet by Compression. By G. P. HACHENBERG, M.D. 869
VII.—Case of Universal Tuberculosis. By J. P. KLUGE, M.D. .......... 872

### BI-MONTHLY ABSTRACTS.

### SURGERY. BY S. W. GROSS, M.D.

A Statistical Examination of the Operation of Deligation of the Iliac Artery.—A Case of Stone in the Bladder.—New Operation for Subunguial Exostosis.—Application of the Button Suture in Varicose Dilatation of the Veins.—On Myeloid Tumors.—Fistulous Ulcer in front of the Larynx.—Treatment of Purulent Ophthalmia of Infants and Children.—Amputation of the Hip-Joint.—Treatment of Stricture of the Urethra.—Fracture of the Surgical Neck of the Scapula.—On a little known Variety of Syphilitic Aphonia.—Treatment of Staphyloma by Ligature .......... 874

PAGE

PATHOLOGY AND PRACTICE OF MEDICINE. BY RICHARD J. DUNGLISON, M.D.

Fatal Case of Trichina Spiralis.—Paralysis of the Bronchial Muscles.—Scrofulous Conformation.—Sudden Death, without Adequate Cause.—Influence of Warm Climates on Phthisis.—Use of the Ophthalmoscope to Diagnose Diseases of the Nervous System.—Intermittent Phenomena, resembling Paralysis.—Habits of Intoxication Causing a Type of Disease.—Progressive Paralysis of the Insane .......... 884

OBSTETRICS, AND DISEASES OF WOMEN AND CHILDREN. BY WM. B. ATKINSON, M.D.

Statistics of Midwifery.—Management of Placenta.—Fœtus carried Twenty-two Months beyond Term.—Can a Woman remain ignorant of her Pregnancy up to Delivery?—Puerperal Convulsions successfully treated with Chloroform.—Treatment of Puerperal Eclampsia.—Ovarian Gestation.—Retained Menses from Imperforate Os Uteri.—Treatment of Vaginitis by Glycerole of Tannin.—Fibrous Tumors of the Uterus.—Simple Instrument for Inflating the Lungs of Infants.—Croup, its Treatment with Antiperiodic Doses of Quinine.—Ophthalmia of New-Born Infants .......... 893

PHYSIOLOGY. BY S. WEIR MITCHELL, M.D.

Anatomy and Physiology; the Reproduction of Bone .......... 904

MATERIA MEDICA AND THERAPEUTICS; MEDICAL CHEMISTRY; TOXICOLOGY AND LEGAL MEDICINE. BY EMIL FISCHER, M.D.

*Materia Medica and Therapeutics:* Kamela; its History, Properties, etc.—Use of Chloride of Zinc in Diseases of the Skin.—Efficacy of some Antipyretic Remedies.—Tinctura Thuiæ in bad Smells of the Nose.—Chlorodyne; its History, Properties, etc.—Opiated Colchicum Wine in Rheumatism.—Caffein as an Antidote to the Poisonous Effects of Opium .......... 911

*Medical Chemistry:* On the Method of Detecting Sugar in Diabetic Urine .......... 918

*Toxicology and Legal Medicine:* Arsenic Eaters of Styria.—On Strangulation... 920

PATHOLOGICAL ANATOMY. BY JOHN H. PACKARD, M.D.

Contributions to Anatomy of the Spinal Cord.—Case of Epilepsy.—Softening of, and Ecchymosis of Blood into, the Pons Varolii.—Cancer of the Heart.—Slit-like Perforation of the Aortic Valves.—Imperforate Arch of the Aorta.—Intussusception of the Ileum.—Stricture of the Rectum.—Abnormal Position of the Right Kidney.—Aneurism of the Internal Iliac.—Nutrition, Inflammation, etc. of Articular Cartilage .......... 927

### BI-MONTHLY PERISCOPE.

SURGERY.—On Coxo-Femoral Disarticulation in Relation to Military Surgery. Report on the Condition of the Prostate in Old Age .......... 939

OBSTETRICS.—On Iodine Injections in Ovarian Cysts .......... 942

PRACTICE OF MEDICINE.—On the Rational Treatment of Delirium Tremens..... 945

EDITORS' TABLE.—The Gold-headed Cane.—Credit to whom Credit is due.—Société d'Anthropologie de Paris.—Changes in Medical Schools.—Medical Journals.—Appointments to the Philadelphia Hospital.—Howard Hospital.—Radical Cure of Consumption.—Introductory Lectures.—American Medical Biography.—Births and Deaths in Philadelphia, in 1859 .......... 948

NECROLOGICAL NOTICES.—Dr. Charles E. Isaacs.—Dr. Charles W. West.—Dr. Addison.—Dr. Robert C. Williams.—Professor Lizars.—Dr. M. S. Buchanan.—Professor Taddei .......... 957

BI-MONTHLY BIBLIOGRAPHICAL RECORD .......... 960

